# Health-seeking behaviour regarding schistosomiasis treatment in the absence of a mass drug administration (MDA) program: the case of endemic communities along Lake Albert in Western Uganda

**DOI:** 10.1186/s12889-023-16020-z

**Published:** 2023-06-05

**Authors:** Maxson Kenneth Anyolitho, Viola Nilah Nyakato, Tine Huyse, Karolien Poels, Caroline Masquillier

**Affiliations:** 1 Department of Community Health, Faculty of Public Health, Lira University, Lira, Uganda; 2 Department of Human Development and Relational Sciences, Faculty of Interdisciplinary Sciences, Mbarara University of Science and Technology, Mbarara, Uganda; 3 Department of Biology, Royal Museum for Central Africa, Tervuren, Belgium; 4 Department of Communication, Faculty of Social Sciences, University of Antwerp, Antwerp, Belgium; 5 Department of Sociology, Faculty of Social Sciences, University of Antwerp, Antwerp, Belgium; 6 Department of Family Medicine and Population Health – FAMPOP, Faculty of Medicine and Health Sciences, University of Antwerp, Antwerp, Belgium; 7 Centre for Family, Population and Health, Faculty of Social Sciences, University of Antwerp, Antwerp, Belgium

**Keywords:** Health-seeking behaviour, Mass drug administration, Endemic, Communities, Lake Albert, Praziquantel, Schistosomiasis, Uganda

## Abstract

**Introduction:**

Schistosomiasis poses a serious public health problem and a social challenge affecting over 240 million people, the majority of whom live in sub-Saharan Africa. The World Health Organization (WHO) recommends praziquantel (PZQ) drug treatment through regular mass drug administration (MDA) accompanied by social mobilisation and health education and sensitisation. With social mobilisation and health education and sensitisation, there is bound to be increased demand for the PZQ, especially in the case of endemic communities. However, it is not clear where communities go for PZQ treatment in the absence of PZQ MDA. We explored the health-seeking behaviours regarding schistosomiasis treatment among communities along Lake Albert in Western Uganda when MDA had delayed, to inform a review of the implementation policy for the achievement of the WHO’s 2030 target of 75% coverage and uptake.

**Methods and materials:**

We conducted a community-based qualitative study in Kagadi and Ntoroko, an endemic community in January and February 2020. We interviewed 12 individuals: local leaders, village health teams, and health workers, and conducted 28 focus group discussion sessions with 251 purposively selected community members. The audio recordings of the data were transcribed and analyzed using a thematic analysis model.

**Results:**

Generally, participants seldom seek medication for schistosomiasis-related signs and symptoms from government hospitals and health centres II, III and IV. Instead, they rely on community volunteers such as VHTs, private facilities, such as clinics and drug shops nearby, or traditional sources (e.g. witch doctors and herbalists). Results show that factors influencing people to seek treatment from sources other than the government are: the absence of PZQ drugs in the government health facility; health workers’ negative attitude towards patients; long distances to the government hospitals and health facilities; poor and inaccessible roads; medication-related costs; and negative perceptions of the PZQ drug.

**Conclusions:**

Availability and accessibility of PZQ seem to be a big challenge. PZQ uptake is further hampered by health systems and community-related and socio-cultural factors. Thus there is a need to bring schistosomiasis drug treatment and services closer to endemic communities, stock nearby facilities with PZQ and encourage endemic communities to take the drug. Contextualised awareness-raising campaigns are needed to debunk myths and misconceptions surrounding the drug.

**Supplementary Information:**

The online version contains supplementary material available at 10.1186/s12889-023-16020-z.

## Introduction

Schistosomiasis is one of the neglected tropical diseases (NTD) that affect a significant proportion of the world’s population and is most prevalent in sub-Saharan Africa [1]. Of all the species, Schistosoma mansoni (intestinal schistosomiasis) is the most common in Uganda [2]. Current control and prevention measures primarily include preventive chemotherapy through mass drug administration (MDA) of PZQ for school-going children aged 5–14 years old and adults in certain situations, supplemented with improvement in water, sanitation and hygiene (WASH), health education and sensitization, and snail control [3, 4]. The WHO categorizes schistosomiasis infection endemic areas into three levels: low for areas below 10%; moderate for those between 10 and 49%; and high for areas from 50% and above [4]. The WHO further recommends that PZQ be administered at least once every 2 years, once a year, and twice a year for low-, moderate-, and high-endemic areas, respectively. Although it is hoped that these interventions can facilitate the achievement of the WHO’s target of 75% MDA coverage by 2030, in Uganda’s case, this is far from attainment. This is evidenced by low uptake among school-going children of 28.2% in 2011 to 48.9% in 2012 and among adult communities of 48.8% in 2016 [5, 6]. To ensure optimum outcomes and sustainability of PZQ uptake, there is a need to improve the MDA implementation strategy to realize increased drug uptake [7]. Moreover, individuals who experience schistosomiasis-related signs and symptoms must always seek PZQ treatment. Some of the signs and symptoms are skin rash, fever, head and body aches, breathing difficulties, diarrhoea and constipation, blood in the feces and swollen bellies [8]. While liver fibrosis, intestinal ulcers, high blood pressure, stunted growth, cognitive impairment in children, and infertility in women appear in advanced stages but are difficult to diagnose [9]. Most of these signs and symptoms show up at different stages after infection, some of which may trigger health-seeking by some community members.

Health-seeking behaviour refers to “any activity undertaken by individuals who perceive themselves to have a health problem or to be ill, to find an appropriate remedy” [10]. Studies conducted on health-seeking behaviour in many parts of the world have suggested that people seek treatment from either biomedical or traditional healthcare sources or both [11]. In Southwest Ethiopia, for instance, most of the urban communities (80.7%) sought health services from modern sources in contrast to rural areas at 48.1% [14]. Determinants of health-seeking also vary across space, time, and different population groups [14]. Factors such as age, sex, education level, marital status, socioeconomic status, social networks, ethnic beliefs, and attitudes among others, have been found to influence the decisions to seek treatment from different sources [14]. Disease-related factors, such as cause and type of illness, acute or chronic illness, and severe or trivial, also influence individuals’ health-seeking behaviour [15]. The above findings suggest that different populations seek medication for certain illness conditions from various sources, but it is not clear whether this is the same for schistosomiasis-related signs and symptoms.

Studies on health-seeking regarding schistosomiasis treatment with PZQ have equally been conducted showing varied findings. For instance, in the Kiri community of Adamawa state- Nigeria, findings showed that a significant proportion of the participants usually bought drugs from the pharmacy or drug shops for schistosomiasis-related signs and symptoms, whereas some got medication from herbalists and the majority never sought medication at all [16]. Likewise in Tanzania, some participants sought medication for schistosomiasis treatment from both traditional and modern healthcare sources, although modern medicine was taken to be the most effective [17]. Findings from studies in Eastern Uganda and Zanzibar, also showed that some participants did not receive PZQ drug treatment because either they were absent or unaware of the MDA program, were busy, feared side effects, or were pregnant [6, 18]. Similarly, in Ethiopia, a study on health-seeking for different NTDs showed that gender disparities in healthcare access and utilization were attributed to inequalities and power dynamics between men and women regarding the decision to seek treatment and its financing [19]. Furthermore, studies show that people with biomedical knowledge of transmission and who perceive the symptoms as severe and acute, tend to seek treatment from modern sources [20, 21]. Lack of health workers and limited drug supplies have also been found to hinder the communities along Lake Victoria in Eastern Uganda from seeking treatment for schistosomiasis-related signs and symptoms from a health facility [22]. This finding was similar to studies in Brazil, the Philippines, and Nigeria where communities reported the following barriers to seeking treatment from a facility: the long distance from the nearest health facility, transport costs, poor road networks, and lack of health information [12, 23, 24]. Whereas these studies have been conducted on health-seeking regarding the treatment of schistosomiasis-related signs and symptoms, they have mainly focused on PZQ of MDA but did not provide an insight into where the populations turn to in the absence of the MDA program.

In Uganda, the PZQ-MDA program has been implemented for almost 20 years now since it was first introduced in 2002 [17]. The PZQ drugs are procured with support from development partners such as the schistosomiasis control initiative (SCI), ARISE and ASCEND, and are managed by the vector control division of the Ministry of Health and delivered to the districts [26]. From the district, the vector control officers together with the district health team distribute them to the communities for VHTs to administer to adult community members and children of five years and above but not going to school, and for teachers to give to school-going children in schools [2]. Before the MDA is implemented, social mobilisation (SM) and health education and sensitization are conducted by the Ministry of Health and donors, that is the development partners [27]. Social mobilisation refers to activities conducted to influence a large number of individuals to take certain actions for the benefit of the community as a whole [28]. In the context of MDA of PZQ, social mobilisation is those activities conducted by the district health team, VHTs, village chairpersons and the development partners, aimed at raising awareness and informing the target communities to participate in the program [29]. Consequently, there has been a steady increase in the uptake of PZQ over the years [6, 26]. SM, in particular, is likely to lead to further increased demand for PZQ drugs. Moreover, endemic communities (especially those that live along the lake shores) that continue to engage in risky water, sanitation, and hygiene (WASH) practices [30, 31], must receive constant treatment to contain the spread of the disease and control morbidity levels. However, in the absence of MDA, it is not clear what such communities do when they need the PZQ. Understanding where the communities go for treatment and the reasons that drive their actions is important to inform policy recommendations for improved NTD control and ultimately eliminate the disease. To investigate this gap in current knowledge and understanding, we conducted a community-based qualitative study among communities in Western Uganda where MDA treatment had not been conducted for more than a year, to explore their health-seeking behaviour regarding schistosomiasis treatment. Specifically, we assessed (1) the communities’ sources of health care services for the treatment of schistosomiasis-related signs and symptoms and, (2) the factors determining their choices of health-seeking regarding schistosomiasis-related signs and symptoms in the absence of the MDA program, to inform government and partners on how to improve praziquantel drug uptake.

## Materials and methods

### Study area and setting

We conducted the study in Kagadi and Ntoroko districts in five sub-counties: Ndaiga, Kyaterekera, Mpeefu and Bwikara and Kanara Town Council. Kanara Town Council and Ndaiga sub-county (mainly fishing communities) are along the Lake Albert shores and are mainly made up of fishermen and fishmongers. The two sub-counties are also where schistosomiasis is highly prevalent. The other three sub-counties are uphill and mostly consist of farming communities. Four of the five sub-counties have health centre III levels, while only Ndaiga has a health centre II, which cannot provide adequate necessary healthcare services like diagnosis and drug treatment for the whole sub-county.

### Study design

The study adopted a qualitative case study design premised on phenomenological theory, to explore endemic communities’ health-seeking behaviour in the absence of the MDA program. We chose this design to describe the health-seeking regarding schistosomiasis treatment in the absence of MDA because it enabled us to capture the communities’ subjective experiences with the signs and symptoms, the actions they take and reasons for their actions in their natural settings [33–34].

### Theoretical framework

**Fig. 1:**
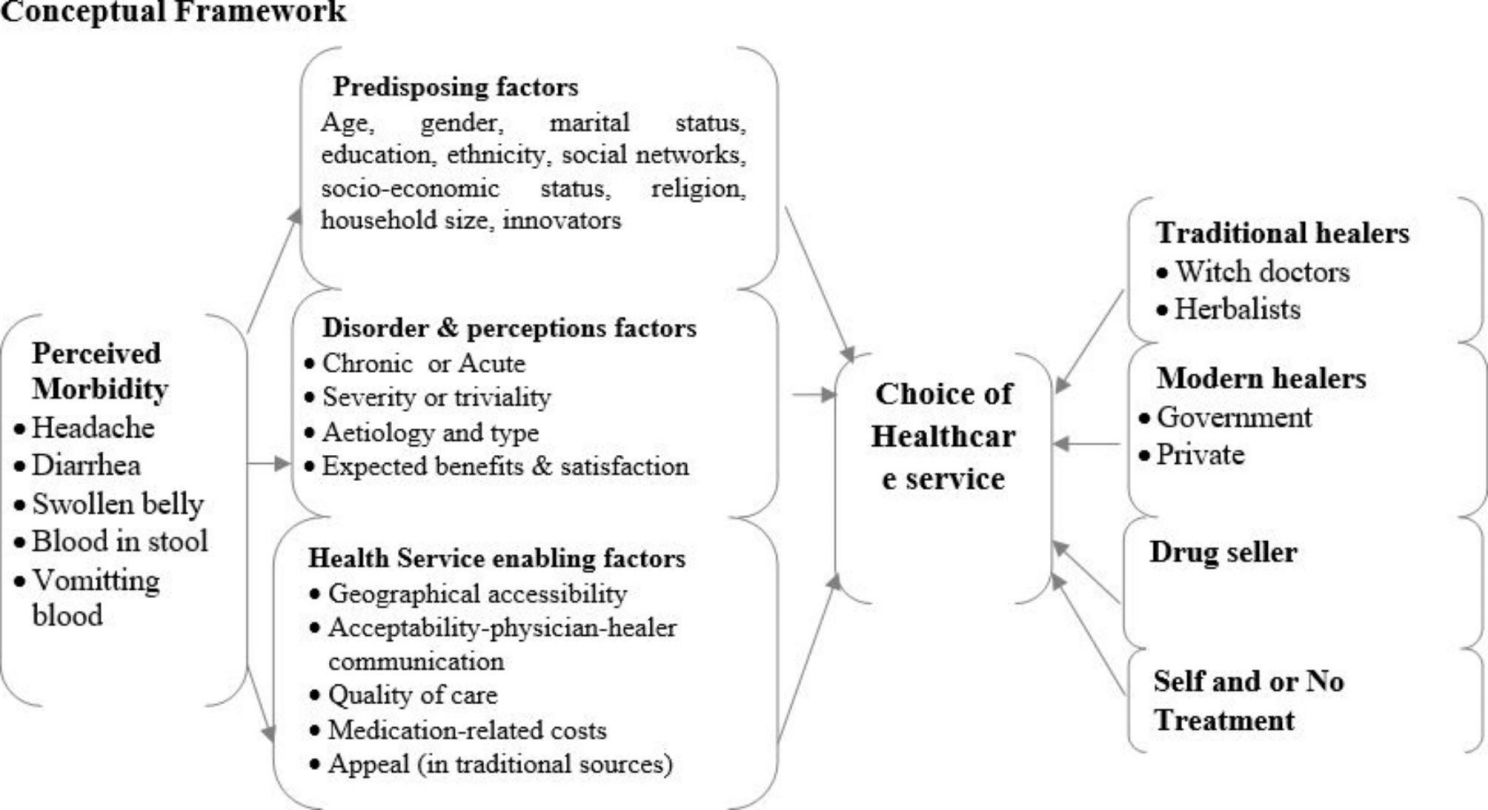
Axel Kroeger’s Model of Health-Seeking Behavior (1983)

The study was based on Kroeger’s theoretical model (Fig. 1). The model states that when faced with morbidities of different kinds, individuals will often decide to seek healthcare services from different sources. The sources include (a)traditional sources (herbalists, witch doctors, and bone setters); (b) modern health services (government hospitals at different levels); (c) private facilities (for-profit or not-for-profit); (d) pharmacies or drug shops; (e) self-medications; and (f) not taking any action at all [36]. According to Kroeger, health-seeking is determined by three sets of factors. First, predisposing factors include age, sex/gender, marital status, household size, education level, social networks and socioeconomic status, and ethnicity, among others [37]. Second, the disorder and the perception that signs and symptoms are chronic or acute, severe or trivial, cause and types, and expected benefits and satisfaction from treatment [14]. Third, enabling factors are accessibility and acceptability, communication between the physician and patient, quality of care, and cost of medication [13]. The model was chosen because it enabled the researcher to holistically examine, analyse and interpret health-seeking actions regarding the treatment of schistosomiasis-related signs and symptoms and the determinants of such actions [35].

### Sample size and participant selection

We estimated a sample size of 295 individuals for the study of whom, 280 were adult community members, while 15 were health workers, village health teams, and local council chairpersons. Estimation was based on the volume of data to be collected, methods of data collection to be used, ease of accessibility of participants, and resource availability [38]. Non-probability purposive sampling technique was used to select participants for the study. The technique was chosen because it enables the selection of participants with characteristics necessary to answer the research questions [32, 39]. The selection of the adult community members was based on their age (adults aged 18 years and above), gender, place of residence, and willingness to participate in the study [32, 40]. Meanwhile, health workers, local leaders and VHTs were selected based on their knowledge about the disease, experience in community mobilisation, health education, and participation in MDA programs and other government-related health programs. The village chairperson together with the VHTs also carry out community mobilisation, while the VHTs do the actual drug distribution and the health workers at district and sub-county levels provide training, coordination of activities, and monitoring and supervision of the exercise [2].

### Data collection

The study employed in-depth interviews (IDI) and focus group discussions (FGD) in the collection of qualitative data from sampled participants. The methods were chosen because they enabled the researchers to triangulate data from participants of different socio-cultural and geographical backgrounds [17]. This way, it was possible to get a nuance of views from the participants that could adequately answer the research question. IDIs were used to collect information on the perspectives of the health workers, VHTs and local leaders regarding health-seeking for Schistosomiasis treatment. Meanwhile, FGDs were employed for the adult community members to explore their experiences of the different signs and symptoms, the actions they take and the reasons behind their actions. Data collection was done by the corresponding author (a male PhD student) and one other master’s female student, both of whom are social scientists qualified and experienced in qualitative research. Before fieldwork, the IDI and FGD guides were pretested to a health worker, VHT and two focus group discussions (eight per session), one for males and another for female community members from a neighbouring sub-county. After the pretest, questions that were not properly constructed were revised, and more probes that came up were added. Mobilisation of IDI participants was done through the sub-county office, while that of FGD participants was done by the chairpersons and VHTs of the respective villages where the study took place. Participants were informed of the date and venue for the exercise in advance. On the data collection day, the researchers took participants through the process of obtaining informed consent. That is, the study objective, participants’ selection procedure, confidentiality, privacy, benefits, risks and willingness to participate were all explained to the participants. Furthermore, permission for audio and video recording and taking pictures was obtained both verbally and in writing. Both the IDIs and FGDs were conducted in the communities but in a quiet place away from noise and people. Audio recordings were done using recording devices upon obtaining permission from participants. On average, the IDI sessions took about one hour, while that of FGDs took about one and a half hours respectively. Data collection took place in English (for the IDIs) and local languages-Alur, Lunyoro, Lutoro and Lunyankole. Where appropriate, local interpreters were employed to help with translation. Key questions asked to the IDI participants were whether they knew where communities go for treatment for schistosomiasis-related signs and symptoms and why. Prompts and probes were made on whether they knew if communities go for PZQ during MDA and where the communities go when the MDA has been delayed. Probes were made to elicit reasons for the choice of the different sources in the absence of MDA and who decides, distance, health workers’ relationships, time spent where they go for treatment, and whether they get the drugs from where they go. Similar questions were posed to the adult community members in the FGD sessions. But this time, participants were asked to share their personal experiences: what actions they take when they experience schistosomiasis-related signs and symptoms, where they go and why with similar probes.

### Data analysis

The study employed a thematic analysis model following Clarke and Braun’s six-step process [41]. First, the corresponding author and the research assistant transcribed the audio recordings, and the corresponding author later uploaded the transcripts into Nvivo V.15 and read through all of them. Next, the corresponding author used both the data collection interview and focus group discussion guide, key concepts from Kroger’s theoretical model (deductively) and a few transcripts (inductively) to develop initial code categories. Third, the corresponding author used the developed code categories to code the remaining transcripts. This was done by highlighting and assigning the different texts to the codes developed earlier on. Where no codes were found, new ones were identified and created from the transcripts to expand the code categories. Next, the coded data were critically examined and sorted to bring similar code categories together and separate those which were different or unique. Fifth, main themes and subthemes were developed from the coded data to suit the research objectives. The major themes generated include modern sources, community volunteers (VHTs and local leaders), private sources, traditional sources and self-medication, pluralist health-seeking, predisposing disorder and perception factors, health services and community factors respectively. Meanwhile, subthemes included government hospitals and health facilities of different levels, clinics, drug shops, witch doctors and herbalists. Also, drug shortage, health workers’ negative attitude, lack of equipment for testing the disease, inadequate staffing, long distance from the facility by some communities, and medication costs and conditions were the other subthemes developed. Finally, we used themes and subthemes to present narratives of the study in a logical manner and supported them with verbatim extracts from the transcripts as evidence.

## Results

### Study participants and their socio-demographic characteristics

Based on the sample size estimated, we contacted 295 individuals for participation in the study. Of this, 29 adult community members either declined to participate or were not present during the FGDs. In addition, two health workers and a VHT who were invited for the in-depth interviews did not turn up. A total of 263 individuals of whom 251 adult community members participated in 28 FGD sessions across the five sub-countries divided by gender (i.e. 14 FGDs for males and another 14 for females). In addition, three health workers, two VHTs, and seven local leaders participated in the in-depth interviews. Furthermore, 134 (50.95%) of the participants were males and 129 (49.05%) were females aged between 18 and 67 years old.

### Sources of healthcare services accessed by participants in the absence of MDA program

This section looks at the first research objective which is on the various health services accessed by communities in Kagadi and Ntoroko Districts in the absence of MDA. Based on Kroeger’s model of health-seeking, qualitative data analysed show that participants seek treatment for schistosomiasis-related illnesses signs and symptoms of different types from different sources which include: modern health services, traditional sources of treatment, and self-medication. Results further show that village health teams and local leaders as community volunteers were the most mentioned sources of health care seeking. This was followed by private clinics and drug shops, together with witch doctors and herbalists, government hospitals and health facilities, self-medication, healer shopping and prayers, with others not taking any action at all as explained below.

#### Community volunteers (VHTs and local leaders)

The majority of participants said they seek PZQ treatment from VHTs and local leaders. Going to this source is usually when the participants start experiencing swollen bellies, blood in stool, vomiting, diarrhoea and fever. The participants said that they go to the VHTs because they think the VHTs should be having the drug since they are the ones who distribute it. Also, the VHTs know about the disease and are therefore in a better position to examine the community members who approach them. It should be noted that VHTs have access to the PZQ only when they are distributing the drug during the MDA program. Sometimes some of the drug stocks remain after the MDA program, and the VHTs give to those who require them. The VHTs also make referrals to health facilities depending on the conditions of the signs and symptoms. According to R 11,

*“They first come to me because they know I always have tablets; after that, I examine the person to see if it’s bilharzia, and if it’s beyond me, I refer them to the health centre” (Male IDI 11, Kanara Town Council).*

#### Private healthcare sources

Equally important was also that, both male and female study participants said they go to private, for-profit facilities more than they do the not-for-profit ones. This was mostly mentioned by those participants who said if they start experiencing the signs and symptoms mentioned above and have the money, they rather go to the clinics. Others, however, mentioned that the absence of drugs and distance from the existing government facilities force them to visit private facilities. This was mainly mentioned by participants from along the lakeshores.

*R10: Yes, I went to a clinic in the village here. The doctor first tested me, and later he told me I had bilharzia and gave me medicine. I don’t know the name of the medicine. That’s the doctor’s job to know the medicine I need, and he gives me the medicine I need to heal. I spent close to 45,000 UGX (approx. 11.00 Euros then) on tests and medicine. (Female FGD 17, Nyamasoga, Ndaiga sub-county)*

#### Traditional healthcare sources

Witch doctors and herbalists are traditional sources that community members rely on. This was reported more by communities that live around lakeshores. Witch doctors invoke the power of the spirits to explain illness, whereas herbalists use herbs, such as tree leaves, shrubs, roots and other parts of plants or animals, to prescribe medication. The main reasons provided by participants for choosing this source of treatment were previous experience with similar signs and symptoms like the swollen belly and blood in stool, perceived severity, ease of accessibility of witch doctors, and misconceptions regarding disease etiology among others.

*“R8: The first thing I think of is getting treatment, in my case, since I have an experience from the previous time when I fell sick or got any other signs I go for, Munganga (witch doctor), because I know it’s a dangerous disease and you can easily die of the disease like the bilharzia*. *R5: Some people may end up visiting witch doctors because of ignorance about the disease. However, some people think perhaps such a disease is a result of eating poison”***(Male FGD-2**, Ndaiga sub-county)

The use of herbs was also reported by some participants living along the lakeshores. The use of herbs was reported to be more for children than adults with emergencies, limited availability of health facilities and the inability to identify the disease being the main reasons. Common signs and symptoms for which the herbs are used include skin rashes, body itching, fever and headache.

*R3: You see, we go to the hospital when we are worse off. We treat the early signs with herbal medicine when they don’t work; that’s when we go to the hospital. We go for herbal medicine because we don’t have an option and we are poor. R4: But what happens here with children is, when they get sick, they give them herbs, and sometimes these herbs work. But if they don’t work, you now go to the hospital. (Female FGD 11, Bwikara sub-county).*

Despite the generally accepted view on their use, other participants reported distrust in traditional sources. They argued that schistosomiasis cannot be cured by witch doctors or by taking herbs, but requires treatments in hospitals and clinics. Distrust in traditional sources of treatment was also compounded by the feeling of worry. To such participants, being infected with schistosomiasis is like a death sentence. That is, there is no cure for it, except waiting to die.

*R7: Suffering from bilharzia, and then you go to the witch doctor? You can’t go to the witch doctor when you have bilharzia. The witch doctors can’t treat it; they will ask you for money and not treat you. .. yes, for other diseases and things, but not bilharzia. (Female FGD 5, Ndaiga sub-county)*

#### Government hospitals and health facilities

The greater majority of participants said they only go to government hospitals for treatment when they start experiencing severe and acute signs and symptoms like swollen bellies and vomiting blood. Meanwhile, they visit health centres II, III, or IV when they experience stomach pain and headaches.

*R10: Yes, I had it before, and even now I have it, as you can see my swollen belly. I had it in 2012, and I got treatment from the hospital. The drug—I don’t know its name—but it looks white. (Female FGD 18, Kanara Town Council)*

*R4: Two years ago, at the Health Centre III, they didn’t get the disease and therefore didn’t give me any drugs. I went because I was feeling unwell; I had stomach pain and thought I had it. So, I said I should first go and check. (Male IDI, Kanara Town Council, Ntoroko)*

#### Self-medication

A few participants reported self-medication. They reasoned that in situations where they face financial challenges, they simply buy some drugs and take them as a temporary measure while they look for money to go to the hospital. Also, when the signs and symptoms are mild, or when the illness attacks at late hours, they just buy drugs or get herbs from nearby available sources. According to R5,

*“If someone falls sick in the night, you just buy Panadol for pain relief and wait until morning. The next morning, if you still feel sick, you can go to the health centre to get tested” (Female FGD 17, Ndaiga sub-county, Kagadi District).*

#### Healer shopping/pluralistic health-seeking and prayers

Still, other participants mentioned seeking treatment from different sources (e.g., clinics), buying drugs, and praying at the same time. The reasons given for such actions include the ease of accessibility of the sources mentioned above and the hospitals being very far. Regarding prayer, participants mentioned that they often pray for themselves or visit pastors to pray for them depending on the circumstances. Also, some people first pray and only try other sources when prayers do not work. Others first start with other sources and only resort to prayers after these sources fail.

*R10: Yes, there are some sicknesses which do not require going to the health centre. Sometimes I go to the hospital; they take my sample and test and don’t find any disease, yet I am not feeling fine, so I go to the pastor, and they pray for me. (Female FGD 21, Kyaterekera sub-county)*

#### Not taking any action at all

Some participants mentioned that they did not take any action whenever they experienced any of the schistosomiasis-related signs and symptoms. The main reasons for this are as follows: the symptoms being mild, the failure to get drugs in the hospitals and facilities, and their failure to carry out diagnostic tests whenever patients go to the health facilities for services. According to R7,

*“The majority suffer in silence, especially if it’s mild—let’s say a simple headache or stomach ache—it’s the minority that seeks medical care” (Male IDI, Ndaiga sub-county).*

### Determinants of health-seeking regarding schistosomiasis related signs and symptoms in the absence of an MDA program

Participants gave several reasons that influenced their decisions to seek medication from the various sources mentioned above. Inspired by Kroeger’s model, we grouped the factors into predisposing or individual factors, disease or disorder- and perception-related factors, health system factors, and community-related factors.

#### Predisposing factors

Kroeger mentions that certain factors predispose individuals to make certain health-seeking decisions, which he terms as predisposing factors. Under this category, we observed in this study gender, marital status and geographical difference, socioeconomic status, and social networks.

##### Gender, marital status and geographical differences

In terms of gender, participants said that generally in a household, the man decides when and where a woman and children should seek treatment. The husband determines the woman’s health-seeking choices regardless of the illness situation. For instance, whenever a woman starts experiencing schistosomiasis-related signs and symptoms such as diarrhoea or abdominal pain or vomiting, she has to first brief the husband of her condition. Afterwards, the man will tell her what next to do, that is, whether she should go to the hospital, just buy drugs or visit a herbalist. Also, it is the husband who provides the money for transport and any needed treatment expenses. Men are culturally obliged to care for their families, and they are heads of families, breadwinners, and income earners who influence their decisions. Finance and responsibility are key factors, as observed through the below quotes.

*R7: It is my husband who decides. R2: It is my husband, just him alone, who decides. R4: I report to my husband and then he is the one to decide. And if he says I go to the government hospital, I will go, and if they prescribe medications, I still ask for money from him. It is he who provides me with the money. (Female FGD 15, Kisenyi B, Kanara Town Council)*

Regarding geographical differences, participants from the lakeshores said that health-seeking decision-making varies from couple to couple, whereas others said the women make their own decisions. Those women who decide for themselves were either divorced or had irresponsible spouses. For such female participants, upon experiencing any signs and symptoms related to schistosomiasis, they just take personal decisions to seek treatment with or without money. In some cases where they don’t have money, the women rely on well-wishers for financial support.

*R6: Our husbands are different, and we are all treated differently; like me, my husband was a drunkard. He didn’t care if I was sick or not. So I had to take myself. R8: You see, most of us here don’t have money. Even if my husband is around, we pass a book around for people to contribute for us—at least 1,000 UGX—so that I can go to the hospital. (Female FGD 17, Nyamasoga, Ndaiga sub-county)*

##### Social networks and socioeconomic status

Social networks, such as family members, relatives, and friends, are relied on for advice on when and where to receive treatment. Women in particular sometimes decide to visit the hospital or get herbs based on their colleagues’ advice upon beginning to feel any signs and symptoms associated with schistosomiasis. In addition, low socioeconomic status also forces some women, especially those from the uphill side, to seek treatment from traditional sources.

According to R3,

*“Here, because we are poor and there’s usually no money, you give the child herbal medicine first, and you only go to the hospital if the symptoms persist” (Female FGD 11, Nyamarembo, Bwikara sub-county).*

#### Disorders and related perception factors

Disorders are how illness signs and symptoms present themselves. Meanwhile, perceptions are the different views and opinions held by people regarding certain signs and symptoms. Both of these are important in deciding whether, when, and where one should go. Under this section and based on Kroeger’s model, subthemes generated include whether the signs and symptoms are acute or chronic and severe or trivial. They also include etiology and type of disorder, satisfaction from treatment, expected benefits and dissatisfaction with service provided, and medication conditions.

##### Acute or chronic and severe or trivial diseases

Participants said generally, they visit the hospital for signs and symptoms (e.g., vomiting blood) that they perceive to be acute. The decision to go to the hospital is further backed by other factors such as distance from the facility, cost of medication, and fear of drug reactions. For other symptoms (e.g., swollen bellies, blood in stool, rushes that are perceived to be chronic), people often normalize them. Trivializing illness conditions are also reported to be common among communities from the lakeshores who argue that they can live with it without any harm. For such participants, treatment is only needed for severe signs and symptoms. Still, other participants argued there is no need to take drugs when they do not feel ill.

*R1: I wasn’t feeling any pain or sickness, so I didn’t swallow them. My wife is the one who swallowed them; she’s the one who was complaining of stomach pain before. R5: I don’t have the disease. (Male FGD 1, Bwikara sub-county)*

##### Etiology and type of disease

Some participants said they seek treatment from traditional sources like witch doctors when they do not know the cause and type of illness, that is, if they cannot tell whether such signs and symptoms are related to schistosomiasis. In situations when they can easily tell that it is something to do with schistosomiasis, they mostly either go to government hospitals or private sources depending on the illness condition. However, this is combined with situations where they perceive the illness to be severe. According to R7,

*“If I don’t know that it’s bilharzia, I would take them to the traditional healers. If it persists, I take them to the hospital” (Male IDI 7, Ndaiga sub-county).*

##### Dissatisfaction with PZQ

Some of the participants, especially those from the lakesides, expressed dissatisfaction with PZQ arguing that whenever they are given the drug, they swallow it, but the drug does not work. The participants said that they still fall sick even after taking the drug. It should be noted that the PZQ drug is given to all eligible people in schistosomiasis endemic areas after getting their age and height but without any diagnosis, with a view that the drug will kill the germ for those who will have had the disease. Therefore, although the drug is used to treat schistosomiasis, it does not necessarily stop a person from getting re-infected, provided that they engage in risky practices. According to R1,

*“The problem is that even the medicine that they give us doesn’t work and people still fall sick” (Female FGD 28, Ndaiga sub-county).*

Coupled with the above is the dissatisfaction with health workers, which was expressed in various ways. Some participants especially those from the lakeshores, complained about how they are served by the facility, the time spent in accessing service, failure to receive service at all, and the unreasonable referral to distant facilities. They observed that whenever they go to government health facilities after starting to experience the above-mentioned signs and symptoms, the health workers simply either look at them or refer them to the main hospitals without saying anything. It should also be noted that PZQ is not provided in government hospitals and lower-level health facilities and neither is it captured in the healthcare system. Some of the participants said thus;

*R10: The nearest health facility is Health Centre II, but if you go there, the health assistants are never there. R: We don’t have doctors who can check for bilharzia in this village. They will refer you to Health Centre III or the referral hospital. But that’s a waste of time because the line is always long and there are no doctors to help. (Female FGD 28, Ndaiga sub-county)*

##### Medical conditions

Some women do not seek treatments for schistosomiasis-related signs and symptoms, giving medical reasons such as being in a first-trimester pregnancy or breastfeeding. The WHO guidelines also prohibit pregnant women, breastfeeding mothers, children below 5 years, and those with certain underlying conditions from taking PZQ. According R3,

*“But they don’t give them to pregnant women. Last year I had taken them, and I was pregnant, but the chairman of this village followed me and took them back from me” (Female FGD 25, Mpeefu sub-county).*

#### Health service–related factors

Kroeger’s model also specifies health service–enabling factors as one of the determinants for individuals’ health-seeking. In this, he combines both of those factors that are health service provider related and those that are community enabling. However, our current study differentiates between health service factors and community factors as we found no enabling factors. Under this particular theme, drug shortage, shortage of health workers, negative attitudes toward health workers, lack of equipment, medication costs, and long waiting times due to work overload are some of the subthemes generated.

##### Drug shortage/inadequate drugs

Drug shortage was highly emphasized as a major factor by participants from both the lakeshores and those on the uphill sides. The participants complained that whenever they visit government facilities, they do not get the schistosomiasis drug. As a result, those with money opt to go to clinics and drug shops, whereas those without money either resort to traditional sources or do not take any action at all.

*R8: But the hospital doesn’t have drugs; they give only one type of drug and send you to another hospital. The problem with government hospitals is that we don’t get drugs when we go to the hospital. But we would love to have the drugs brought here. (Female FGD 18, Kanara Town Council)*

##### Shortage of healthcare workers

Also observed by participants was the limited number of staff in most government facilities such as hospitals, and health centres IV, III, and II. Participants argued that whenever they go for services at these facilities, they often do not find staff that can attend to them, thereby discouraging them from seeking services from the facilities.

*R1: We never find the doctors who test for bilharzia. Unlike HIV, where you can even be tested in a clinic, you can’t find doctors to test you for bilharzia. On top of that, we have few doctors, yet there are many patients. That means sitting the whole day to see a doctor. All the nearby clinics will refer you to the health centre III. R4: You see, even if you go all the way to Bwikara Health Centre, there’s no doctor to test for bilharzia. (Male FGD 1, Bwikara sub-county)*

##### Negative attitudes of healthcare workers

Participants, particularly the female ones, spoke about how health workers, especially from government facilities, handled them. The participants complained that whenever they visit the facilities, health workers do not properly attend to them. Sometimes staff ignore them and do not talk to them.

*R2: Even if you go to the health centre, they just look at you. I was once sick, but when I went there, they just looked at me. I was suffering from a headache. Then I went to the clinic and bought medicine. (Female FGD 21, Kyaterekera sub-county)*

##### Waiting time

The long waiting time for health workers to serve them was also highlighted as a hindering factor. Still, health workers simply refer them to other facilities after seeing but not talking to them. To make matters worse, they are not given any treatment, and no reasons are provided for the referral. According to R2,

*“On top of that, we have few doctors, yet there are many patients. That means sitting the whole day to see a doctor. All the nearby clinics will refer you to a health centre III” (Male FGD 1, Bwikara sub-county).*

The community’s views were supported by the VHTs and local leaders who said that PZQ tablets are not supplied in time, are inadequate, usually target children of school-going age, and do not cover every village.

*R11: But right now, I don’t think there is bilharzia medicine because I got a case that I referred to the health in-charge, and he said there was no medicine he had to first call the district to get the medicine here. (Male IDI, Kanara Town Council)*

##### Medication costs

Participants also reported the high medication cost as a hindrance to health-seeking, especially from private, for-profit facilities such as clinics and drug shops. Participants said that it is costly to get treatment for schistosomiasis illnesses from private facilities.

*“R9: We have clinics, but if you don’t have the money, you can’t go there. The clinics are for people with money. For testing, it’s usually 20,000 UGX (Approx 5.00 Euros then), and then for pregnant people, going to the scan is 10,000 UGX (Approx 2.00 Euros then). Then medicine can cost you roughly 20,000 UGX (Approx 5 Euros then) depending on what you are suffering from. But if you are going to a clinic, you can prepare in total 50,000 UGX (Approx 12.50 Euros then).” (Female FGD 28, Ndaiga sub-county)*

##### Lack of equipment for testing schistosomiasis

Participants also mentioned a lack of equipment, which makes it difficult for the health facilities to carry out certain diagnostic tests, and is another factor hindering health-seeking from modern sources. This is because lack of diagnosis discourages communities from seeking medications from facilities. According to R1,

*“There is no machine to do that. Even if you go to the main hospital, you can’t find it” (Male FGD 24, Mpeefu sub-county).*

#### Community-related factors

Community-related factors are conditions outside the individual’s and health service providers’ control, and they are not disorder related. Such factors can inhibit individuals from seeking services from modern sources. Kroeger’s model had indicated these factors as enabling rather than inhibiting. Subthemes here include the long distance from facilities, high transport costs and poor and inaccessible roads, a long stay in the lake waters while fishing, and migration from one landing site to another.

##### Long distance from facilities

Long distance was a concern mainly mentioned by the majority of participants from the lakeshores. They explained that the facilities are situated very far from the communities, thereby forcing them to walk for several hours. According to R1,

*“It’s a seven-hour walk from here to the Health Centre, and if you start walking at 6 a.m., you can reach it by midday” (Female FGD 28, Ndaiga sub-county).*

##### High transport cost

High transport cost was another concern raised by participants, especially those at the lakeshores. Participants noted that due to bad roads, motorists often charge a lot of money to take them to the nearest facilities. Although water transport would be an alternative for those along the lakeshores, it was equally reported to be costly, hence hindering accessibility to modern healthcare services.

*R4: Like, for my case, I go to the hospital because they have the machine for testing it, and it is just near. We use water transport (canoe), and it is near enough to our place for us to go to Ntoroko. We pay 10,000 shillings to and from. (Male FGD 4, Ndaiga sub-county)*

##### Poor and inaccessible roads

Another factor mentioned by participants was the poor and sometimes inaccessible road network. A local leader expressed concern that poor and inaccessible roads make it difficult for communities to seek medication from health facilities, hence leading to maternal and infant mortality.

*R6: I would ask the government to bring the services closer to us because we have some maternity women who cannot even move; for example, yesterday when I was going to the hospital, I found pregnant women climbing the hill. The health centre and main government hospital are far from this village. (Male IDI, Ndaiga sub-county)*

##### Long stay in the Lake and migration from one landing site to another

For some participants at the lakeshore, the nature of their fishing—whereby they move from one landing site to another in search of “better fishing grounds”—also causes them to miss the MDA programs. Furthermore, some of them often go deep into the lake to carry out fishing activity, which sometimes takes a month. Thus, they end up missing the PZQ drug. According to the guidelines, a person should be physically present, so their height is taken and the drugs are given. This becomes a challenge for such categories of people, as stated here by R6,

*“Yes, indeed, some people don’t get it because they are always in the water and doing some fishing activities; also, some are working and do miss the time of taking their drugs” (Male IDI, Ndiaga sub-county).*

## Discussion

Health-seeking behaviour regarding schistosomiasis treatment using PZQ MDA has been widely researched across the globe [12, 42]. However with social mobilisation, the likelihood of demand for PZQ increases. It is therefore important to properly understand where people seek treatment and the factors that influence their health-seeking when there is no MDA program or when it has been delayed. We conducted a community-based qualitative study to explore the health-seeking behaviour of the endemic communities of Kagadi and Ntoroko Districts in Western Uganda, at a time when the routine MDA program had been skipped. The study was guided by Kroeger’s model of health-seeking behaviour. This section presents a discussion of the study findings.

Regarding sources of health-seeking, our current study found that VHTs are the main source where people seek treatment for schistosomiasis-related signs and symptoms. This is possible because VHTs are known by the communities for distributing PZQ during MDA and so, they are thought to always have the drug [43]. Sometimes, some stock remains with the VHTs after the exercise and so they give it to those who ask for it. Most times, however, the drugs get finished during the MDA program. VHTs are also the first point of contact for those with signs and symptoms of the disease. Strengthening the community-based volunteer approach to drug distribution by increasing the number of VHTs, adequately facilitating them and giving them more powers to manage the MDA, could go a long way in ensuring the drugs are easily accessible and owned and managed by the communities for sustainability purposes. Results from the study further revealed that the absence of PZQ from the VHTs and government facilities forces communities to seek treatment from private facilities, while others go to traditional sources for mild conditions or other unexplainable ones. Seeking treatment from sources other than modern is likely to compromise the efforts to increase PZQ uptake. Our findings resonate well with a study in Eastern Uganda that also showed people seek health services from various sources for different reasons, with some of them going to traditional sources. However, this was reported to be mainly due to a lack of knowledge of the disease and long distances from health facilities, but not drug absence [2, 6, 24, 26].

Our current study also found that gender and geographical differences among others determine the community’s health-seeking behaviour regarding schistosomiasis signs and symptoms, especially in the absence of the MDA program. Results show that female community members often ask their spouses whether and where they should seek treatment for schistosomiasis-related signs and symptoms. This is likely to be true because of the cultural beliefs that attribute decision-making to men in these communities. Understanding gender factors is crucial for planning, designing, and implementing treatment and awareness campaign programs because it facilitates the inclusion of both men and women. A similar study also revealed that gender norms contribute to women and children missing medication while fetching water, washing clothes and utensils and men fishing, even though the study focused on the MDA program only but not in its absence [44]. Also, studies on other diseases instead linked women’s lack of decision-making to the stigma associated with some of the diseases, shame, and discomfort, among others [19]. This could be possible because of the different perceptions and interpretations of various diseases. Further, in this study, those from along the shores tend to take swift action to go to the drug shops or visit witch doctors and consult herbalists more often than those from the uphill. This is possibly due to the lack of health facilities nearby and the fact that witch doctors and herbalists live within the communities.

Concerning disorder- and perception-related factors, our current study found that, for acute and severe signs and symptoms of schistosomiasis, people go to government hospitals. While those signs and symptoms perceived to be chronic and less severe are not acted upon. Meanwhile, lower health facilities, herbs, or witch doctors are turned to for conditions for which the cause and type of illness are not known. This is most likely due to the absence of drugs and poor health services, leaving the communities with no choice but to try those alternative sources mentioned. But this could also be attributed to myths and misconceptions held by the communities regarding the disease. Myths and misconceptions were reported by another study to be affecting MDA uptake [45]. Other studies have also indicated that health-seeking from either modern or traditional sources is influenced by the perceived severity of signs and symptoms [24, 42, 46].

Regarding health service factors, we found that long waiting time, health workers’ negative attitudes, drug shortage, lack of equipment, and high cost of medication hinder health-seeking from modern sources. This could explain the community’s choice of health-seeking from alternative sources such as clinics, drug shops, witch doctors, and herbalists. Addressing some of these challenges could probably motivate communities to seek treatment from modern sources, thereby increasing trust in the PZQ and its uptake. Our study is supported by findings from a study in Eastern Uganda that demonstrated that institutional factors like inadequate preparation of children and teachers and facilitation of teachers impede biomedical health-seeking [25].

Community-related factors, such as long distances, poor and inaccessible roads, and high cost of transport, were most pronounced among all other factors influencing participants’ health-seeking behaviour. This can discourage communities from going to health centres and hospitals, which could explain why people resort to nearby sources. Our current study is inconsistent with a study in Ghana that found that the perceived severity of disease, rather than long distance, was the main driver of health-seeking from modern sources [7]. Possible reasons for variations could be due to the difference in geographical settings whereby our study setting had some communities that are geographically hard to reach, while the setting in Ghana could be different.

**Fig. 2:**
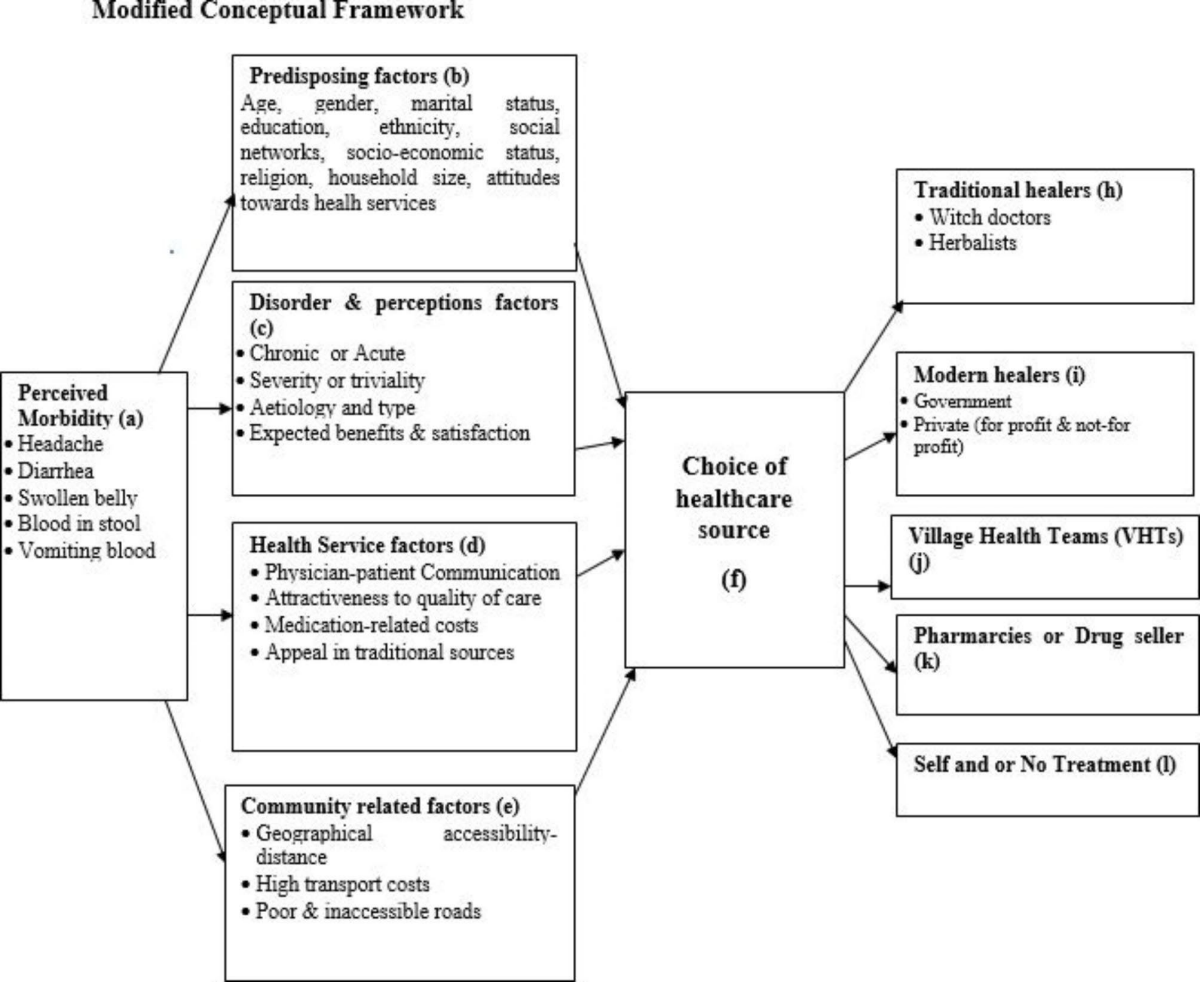
Modified version of Alex Kroeger’s model of health-seeking behaviour (1983)

Finally, we found that Kroeger’s model (Fig. 1) has been relied upon or modified by several other studies to explain illness health-seeking behaviour generally but not specifically concerning Schistosomiasis [10, 14, 37, 47–49]. However, we found that the model could not fully explain certain aspects of health-seeking regarding schistosomiasis treatment in the absence of MDA. First, it could not explain the use of VHTs as another source that communities have known for a long time in distributing the PZQ. And so they believe that the VHTs should be having the drugs, even when the VHTs also ran out of stock. In addition, Kroeger combines both health service and community factors into a single set of factors called health service–enabling factors. This makes it difficult for a reader to understand their interpretations. It would be better if such factors were looked at separately. We, therefore, propose a modified version of the model that integrates the VHTs as another source of health-seeking regarding schistosomiasis treatment by endemic communities, while health service and community factors are treated separately (Fig. 2). We believe that this modified model will help further explain health-seeking regarding the illness that requires, in particular, the involvement of volunteers (e.g., VHTs), as well as explain where people seek treatment when there is no MDA and the reasons behind their choices so that appropriate actions are taken.

## Conclusions

Our current study provides interesting insights into the study of health-seeking behaviour regarding schistosomiasis treatment in the absence of MDA, to inform policy recommendations. In this study, we have demonstrated that health-seeking regarding the treatment of schistosomiasis-related signs and symptoms by endemic communities is likely to be a challenge when the MDA of PZQ is not in place. There is, therefore, a need to bring PZQ drug treatment closer to endemic communities and ensure its constant availability. Furthermore, active community involvement in the planning and implementation of treatment programs is key to the achievement of tangible outcomes. Traditional health practitioners and private health facility operators, in particular, need to be educated and sensitized about schistosomiasis prevention and treatment and, where possible, collaborate with them to address the existing barriers to health-seeking. Communities must be encouraged to seek treatment for schistosomiasis-related signs and symptoms from modern sources and to take PZQ when infected. Future research could explore the potential of integrating PZQ treatment into the mainstream healthcare system and also with a focus on WASH-related interventions using a bottom-up approach. Finally, this current study relied on the communities’ subjective views on health-seeking regarding the treatment of schistosomiasis-related signs and symptoms which could have introduced participants’ biases that might have affected the findings and conclusions. In future, we recommend further research to find out the facilities’ perspective on the availability, access, affordability and utilisation of PZQ for the treatment of schistosomiasis-related signs and symptoms.

## Electronic supplementary material

Below is the link to the electronic supplementary material.

Supplementary Material 1

Supplementary Material 2

## Data Availability

The datasets generated and/or analyzed during the current study are not publicly available but can be accessed upon reasonable request from the corresponding author.
