# Supplementary material for: Health-seeking behaviour regarding schistosomiasis treatment in the absence of a mass drug administration (MDA) program: the case of endemic communities along Lake Albert in Western Uganda
Source: BMC Public Health. 2023 Jun 5;23:1072. doi: 10.1186/s12889-023-16020-z (PMC10240754; doi:10.1186/s12889-023-16020-z)
Supplement: Supplementary file 1 — Supplementary Material 1 [file 12889_2023_16020_MOESM1_ESM.doc]

***Topic:*** ***An exploratory study of the health-seeking behaviour regarding schistosomiasis treatment in the absence of a mass drug administration program among endemic communities of western Uganda***

**IN-DEPTH INTERVIEW GUIDE FOR WITH SELECTED COMMUNITY LEADERS (Health workers, Local council chairpersons, Village Health Teams-VHTs, etc)**

1. **Introduction**: The interviewer/researcher introduces the team, the research and the purpose of the interview
2. **Demographic Information-**I would like to know your age, gender, level of education, working status, marital status and nearest health facility from your home
3. **Sources of health seeking:**
   1. What schistosomiasis-related signs and symptoms do you or your close relatives experience in this community? (probe for the swollen belly, blood in stool, diarrhoea, headaches, skin rash, body itches etc).
   2. For those signs and symptoms**,** how do you experience them? (probe for how they come about, acute or trivial, perceived severity, etc)
   3. Government usually distributes praziquantel (PZQ) in this community, but sometimes it is delayed, in short supply or not provided at all. In such situations, where do you or your close relatives go for treatment when you experience the above signs and symptoms? *(hospitals whether government or private-clinic, pharmacies/drug shops, village health teams-VHTs, traditional sources-herbs, witchdoctors, prayers, or no action)?*
4. **Determinants of Health Seeking regarding schistosomiasis treatment**
   1. For those places mentioned above, what are some of the reasons you or your close relatives would give for seeking treatment? *(probe distance, health workers' attitudes, drug stock out, limited staff, long time taken while accessing services, finance, poor roads, transport-related challenges, marital status etc)*
   2. If you or your close relatives do not take any action, why could that be? (probe for different reasons-perceived lack of knowledge of the illness, advice from relatives etc)
   3. Gender dynamics and complexities**-**Who in your family decides what should be done when you or any of your close relatives start experiencing schistosomiasis-related signs and symptoms and why?
5. We are now coming to the end of our interaction. Do you have any other things else to share with us regarding this topic?
6. **Conclusion:** Thank you so much for taking the time to participate in this study. I will be happy to answer any questions or respond to any comments from you.

***Thank you very much the conversation has ended.***
