# Supplementary material for: Health-seeking behaviour regarding schistosomiasis treatment in the absence of a mass drug administration (MDA) program: the case of endemic communities along Lake Albert in Western Uganda
Source: BMC Public Health. 2023 Jun 5;23:1072. doi: 10.1186/s12889-023-16020-z (PMC10240754; doi:10.1186/s12889-023-16020-z)
Supplement: Supplementary file 2 — Supplementary Material 2 [file 12889_2023_16020_MOESM2_ESM.doc]

***Topic: An exploratory study of the health-seeking behaviour regarding schistosomiasis treatment in the absence of a mass drug administration program among endemic communities of western Uganda***

**FGD GUIDE WITH SELECTED COMMUNITY MEMBERS FOR KAPs STUDY**

1. **Introduction:** The research team, purpose of the meeting including some brief background to the study
2. **Demographic information**

- I would like us to start by getting some information on your age, gender, level of education, occupation, and marital status just for personal records purposes.

1. **Sources of health seeking:**

We are now going to discuss something about the signs and symptoms related to schistosomiasis that people in this community experience, how they come, what they do with it and where they go and why.

- 1. Can you briefly mention for me the schistosomiasis-related signs and symptoms you or your close relatives in this community experience? (probe for the swollen belly, blood in stool, diarrhoea, headaches, skin rash, body itches etc).
  2. I would like you to share with me how you experience those signs and symptoms mentioned above. That is, how they come about, whether they are acute or chronic, severe or trivial, etc)
  3. We understand that the government usually distributes praziquantel (PZQ) drugs in this community, but sometimes it is delayed, in short supply or not provided at all. Now in the above circumstance of the absence of praziquantel, I would like to know from you people, where do you or your close relatives go for treatment when you experience the above signs and symptoms? *(hospitals whether government or private-clinic, pharmacies/drug shops, village health teams-VHTs, traditional sources-herbs, witchdoctors, prayers, or no action)?*

1. **Determinants of Health Seeking regarding schistosomiasis treatment**
   1. Would you please tell me the reasons you or your close relatives would give for seeking treatment for schistosomiasis signs and symptoms mentioned above? *(probe distance, health workers' attitudes, drug stock out, limited staff, long time taken while accessing services, finance, poor roads, transport-related challenges, marital status etc)*
   2. Now for those who do not take any action at all upon experiencing the above signs and symptoms, what could be the reason? (probe for different reasons-perceived lack of knowledge of the illness, advice from relatives etc)
   3. Gender dynamics and complexities**-**Who in your family decides what should be done, or where you should go when you or your close relatives including young ones start experiencing schistosomiasis-related signs and symptoms and why?
2. We are now coming to the end of our interaction. Do you have any other things else to share with us regarding this topic?
3. **Conclusion:** Thank you so much for taking the time to participate in this study. I will be happy to answer any questions or respond to any comments from you.

***Thank you very much the meeting has ended.***
